# Identification by virtual screening and functional characterisation of novel positive and negative allosteric modulators of the α7 nicotinic acetylcholine receptor

**DOI:** 10.1016/j.neuropharm.2018.07.009

**Published:** 2018-09-01

**Authors:** Charles L.C. Smelt, Victoria R. Sanders, Joseph Newcombe, Richard P. Burt, Tom D. Sheppard, Maya Topf, Neil S. Millar

**Affiliations:** a Division of Biosciences, University College London, London, UK; b Department of Chemistry, University College London, London, UK; c Institute of Structural and Molecular Biology, Birkbeck College, London, UK

**Keywords:** Nicotinic acetylcholine receptor, Ion channel, Allosteric modulation, Virtual screening, 5-HT, 5-hydroxytryptamine, DB04763, 1-N-(4-sulfamoylphenethyl)-2,4,6-trimethylpyridinium tetrafluoroborate, AUC, area under the curve, CAII, carbonic anhydrase II, CDK2, cyclin-dependent kinase 2, DB08122, *N*-methyl-4-{[(2-oxo-1,2-dihydro-3h-indol-3-ylidene)methyl]amino}benzenesulfonamide, GABA, γ-aminobutyric acid, NAM, negative allosteric modulator, nAChR, nicotinic acetylcholine receptor, NKCC, Na^+^-K^+^-Cl^-^ cotransporter, PAM, positive allosteric modulator, ROC, receiver operator characteristic

## Abstract

Several previous studies have demonstrated that the activity of neurotransmitters acting on ligand-gated ion channels such as the nicotinic acetylcholine receptor (nAChR) can be altered by compounds binding to allosteric modulatory sites. In the case of α7 nAChRs, both positive and negative allosteric modulators (PAMs and NAMs) have been identified and have attracted considerable interest. A recent study, employing revised structural models of the transmembrane domain of the α7 nAChR in closed and open conformations, has provided support for an inter-subunit transmembrane allosteric binding site (Newcombe et al 2017). In the present study, we have performed virtual screening of the DrugBank database using pharmacophore queries that were based on the predicted binding mode of PAMs to α7 nAChR structural models. A total of 81 compounds were identified in the DrugBank database, of which the 25 highest-ranked hits corresponded to one of four previously-identified therapeutic compound groups (carbonic anhydrase inhibitors, cyclin-dependent kinase inhibitors, diuretics targeting the Na^+^-K^+^-Cl^-^ cotransporter, and fluoroquinolone antibiotics targeting DNA gyrase). The top-ranked compound from each of these four groups (DB04763, DB08122, furosemide and pefloxacin, respectively) was tested for its effects on human α7 nAChR expressed in *Xenopus* oocytes using two-electrode voltage-clamp electrophysiology. These studies, conducted with wild-type, mutant and chimeric receptors, resulted in all four compounds exerting allosteric modulatory effects. While DB04763, DB08122 and pefloxacin were antagonists, furosemide potentiated ACh responses. Our findings, supported by docking studies, are consistent with these compounds acting as PAMs and NAMs of the α7 nAChR via interaction with a transmembrane site.

## Highlights

• Identification of α7 nAChR positive and negative allosteric modulators.
• Furosemide is a positive allosteric modulator of α7 nAChRs.
• DB04763, DB08122 and pefloxacin are negative allosteric modulators of α7 nAChRs.
• Modulation of α7 nAChRs by an allosteric transmembrane site.

## 1 Introduction

Nicotinic acetylcholine receptors (nAChRs) are members of the superfamily of pentameric ligand-gated ion channels, that also includes receptors for 5-hydroxytrptamine (5-HT), γ-aminobutyric acid (GABA) and glycine (Changeux, 2012). Seventeen nAChR subunits have been identified in vertebrates (α1-α10, β1- β4, γ, δ and ε) that can co-assemble to generate a diverse family of pharmacologically distinct nAChR subtypes (Millar and Gotti, 2009). The human α7 nAChR has attracted interest as a target for therapeutic drug discovery, which has arisen, in part, from evidence that α7 nAChRs may play a role in a range of neurological and psychiatric disorders (Parri et al., 2011; Wallace and Porter, 2011). In particular, considerable attention has focussed on studies of positive allosteric modulators (PAMs) that are thought to bind within the receptor's transmembrane domain (Williams et al., 2011; Chatzidaki and Millar, 2015).

The nAChR orthosteric binding site is located in the extracellular domain, at the interface between subunits (Changeux, 2012). Therefore, we consider allosteric binding sites to be any binding site that is topographically distinct from the binding site of the endogenous agonist (the orthosteric site). In addition to PAMs, which are allosteric ligands that potentiate agonist-evoked responses, negative allosteric modulators (NAMs) reduce agonist-evoked responses. Homomeric α7 nAChRs are characterised by their relatively low ACh sensitivity, rapid activation and fast desensitisation (Couturier et al., 1990). By convention, α7 nAChR PAMs have been classified as either ‘type I’, which have little or no effect on desensitisation kinetics, or ‘type II’, which reduce the rate of receptor desensitisation (Bertrand and Gopalakrishnan, 2007). However, there is also evidence for α7-selective PAMs with intermediate properties (Chatzidaki et al., 2015).

We have recently generated revised structural models of the human α7 nAChR, based on the cryo-EM structure of the *Torpedo* electric organ nAChR in its closed and open conformations, in which an error in the transmembrane domain of the *Torpedo* nAChR structure has been corrected (Newcombe et al., 2017). Previous computer docking studies performed with our revised human α7 nAChR structural models identified an inter-subunit transmembrane site for allosteric modulators (Newcombe et al., 2017). In the present study, we have extended these findings by generating pharmacophore models to perform virtual screening of the DrugBank database (Wishart et al., 2006). DrugBank is a relatively small database, containing approximately 11,000 compounds that act on identified drug targets, of which a relatively high proportion (approximately 2500) are approved small molecule drugs. Our goal in performing virtual screening with pharmacophore queries based on a series of known α7 nAChR PAMs was to identify compounds that may interact with the predicted allosteric transmembrane site and may therefore act as α7 nAChR allosteric modulators.

All of the 25 highest-ranked hits identified by virtual screening were compounds that are known to act as inhibitors of one of four previously identified protein targets: carbonic anhydrase II (CAII), cyclin-dependent kinase 2 (CDK2), Na^+^-K^+^-Cl^-^ cotransporter (NKCC) and DNA gyrase (DNAG). Drugs acting on these protein targets have been developed for use in the treatment of glaucoma (CAII inhibitors), as anti-cancer therapies (CDC2 inhibitors), as diuretics (NKCC inhibitors), or as antibiotics (DNA gyrase inhibitors). The highest ranked compounds identified by virtual screening from each of these four drug groups (DB04763, DB08122, DB00695 [furosemide] and DB00487 [pefloxacin], respectively) were tested for their effects on human α7 nAChR expressed in *Xenopus* oocytes. By means of two-electrode voltage-clamp recording, all four of the compounds were observed to have either positive or negative modulatory effects on α7 nAChRs, either potentiating or antagonising responses to acetylcholine. Three of the compounds (DB04763, DB08122 and pefloxacin) were found to act as NAMs of the α7 nAChR, whereas furosemide was an α7 nAChR PAM. The findings provide strong and direct evidence that virtual screening can be an effective approach for the identification of compounds with allosteric modulatory effects on neurotransmitter receptors such as the nAChR, even when employed with relatively small compound libraries.

## 2 Materials and methods

### 2.1 Virtual screening

**Fig. 1:**
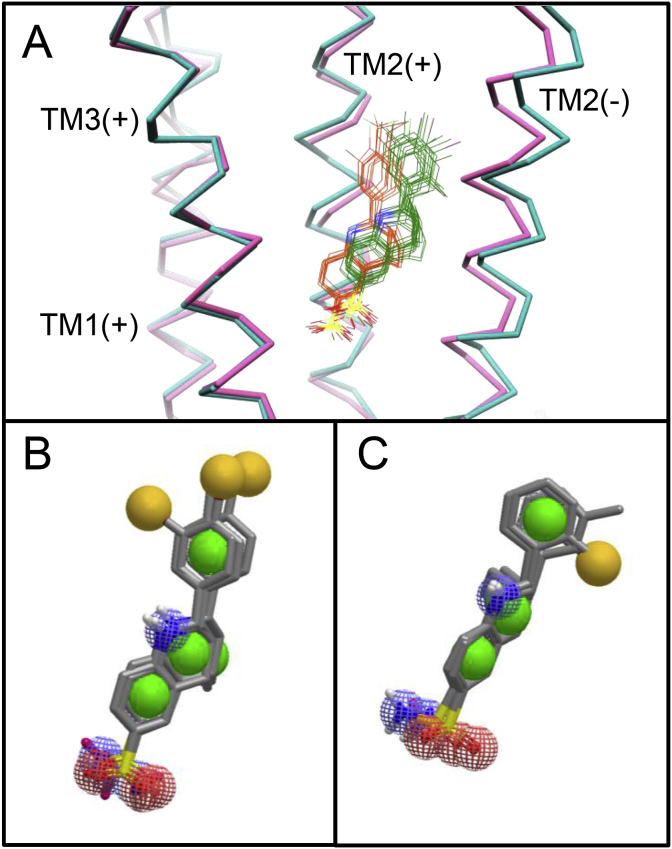
Generation of pharmacophore queries used for virtual screening. The highest ranked clusters of binding mode solutions with previously characterised PAMs are shown within the α7 nAChR transmembrane domain (A). The Cα trace of TM1-3 helices of the principal subunit and TM2 helix of the complimentary subunit are shown for the open (cyan) and closed (pink) conformations. Also shown are binding mode clusters from which pharmacophore queries were generated for the open (green) and closed (orange) conformations. From the ligands in each cluster, pharmacophore queries were generated for the closed and open conformations (B and C, respectively). Note, only those selected for screening are shown. Features of the pharmacophore are represented as yellow spheres (hydrophobes), green spheres (rings), red hashed spheres (hydrogen bond acceptors) and blue hashed spheres (hydrogen bond donors). (For interpretation of the references to color in this figure legend, the reader is referred to the Web version of this article.)

**Fig. 2:**
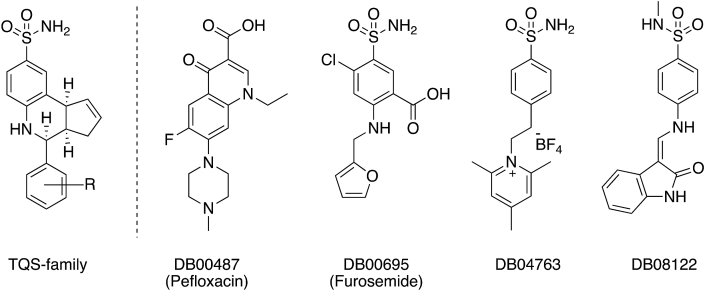
Allosteric modulators of the α7 nAChR. The general structure of the ‘TQS-family’ of compounds that were used to generate the phamacophore query is shown on the left. Four compounds from the DrugBank database that were identified by virtual screening and selected for functional characterisation are shown on the right.

A group of 25 α7 nAChR PAMs sharing close chemical similarity were selected (see the representative ‘TQS-family’ structure illustrated in Fig. 2 and also the compounds identified as ‘TQS-family’ in the supplemental Table 1 of Newcombe et al., 2017). These compounds were docked into revised structural models of the α7 nAChR transmembrane domain in both the open and closed conformations (Newcombe et al., 2017). Using a previously described consensus docking protocol (Newcombe et al., 2017), the top five solutions for each of the PAMs were clustered by RMSD with a cut-off of 2.0 Å. The largest cluster found for each of the open and closed docking experiments was taken to represent the active conformation of the ligand in each receptor conformation (Fig. 1). Three 3D pharmacophore queries were created based on each of the two clusters (one from the open form and the other from the closed form of the α7 nAChR structural model). This was done using the ligand model builder tool from the software package Rapid Overlay of Chemical Structures (ROCS) (Rush et al., 2005), allowing a maximum of six ligands to be utilized by the query generation algorithm. ROCS built every variation of possible query models containing between one and six ligands from the supplied binding mode cluster, creating a gaussian volume corresponding to the molecular shape of the overlaid ligands and assigning ‘color atoms’ at pharmacophoric points associated with hydrogen bond donors, hydrogen bond acceptors, rings and hydrophobes in the ligands that contributed to each of the queries that were built. Every built query was screened against the ligands in the cluster and the three queries with the highest average similarity to all the ligands from the cluster determined by the Tanimoto Combo score (Tanimoto, 1958) for both open and closed conformation binding mode clusters were selected for testing in validation screening runs (Fig. 1B and C). Validation runs were carried out in which the pharmacophore queries were screened against a database containing 42 known active α7 nAChR PAMs (Table S1) from which those PAMs used to generate the query had been excluded) and a set of likely non-binders, or decoys (36 decoys for each known active compound), generated with decoy finder (Cereto-Massagué et al., 2012). The decoy set was generated by taking a subset of compounds from the ZINC database (Irwin and Shoichet, 2005), ensuring that the Tanimoto coefficient (Tanimoto, 1958) between the ligands in the known binder set and the decoy sets did not surpass a threshold of 0.8. For the two sets of 3D pharmacophore queries, the query with the largest area under the curve (AUC) in its receiver operator characteristic (ROC) plot and with the highest early enrichment factor was chosen to use for screening (Table S2 and Fig. S1). Next, the DrugBank database was screened to identify potential new drug-like ligands for the α7 nAChR. Two filters were applied to the top 100 hits from each query, as determined by the Tanimoto Combo score. The first filter compared open and closed conformations of each group of compounds, with only compounds that appeared in both hit lists being selected. The second filter was applied by selecting only compounds for which the central nervous system multi-parameter optimisation score was 4.0 or above. This score was calculated with an in-house script based on the central nervous system multi-parameter optimisation (Wager et al., 2010, 2016) using ChemAxon calculator plugins (ChemAxon, Budapest, Hungary) to predict the physico-chemical properties of the small-molecules.

### 2.2 Compounds and chemical synthesis

DB04763 and DB08122 were synthesised by methods that have been described previously (Supuran et al., 1998; Bramson et al., 2001) full details are provided in the supplementary data. Reagents for chemical synthesis were purchased from Fluorochem (Hadfield, UK) or Sigma-Aldrich (Gillingham, UK). All other compounds, including furosemide and pefloxacin, were purchased from Sigma-Aldrich (Gillingham, UK).

### 2.3 Plasmids and site-directed mutagenesis

Oocyte expression studies (see below) employed the human α7 nAChR subunit in plasmid pSP64GL (Broadbent et al., 2006), the mouse 5-HT_3A_R subunit in pRK5 (Maricq et al., 1991), and a human α7 nAChR/mouse α7/5-HT_3A_R chimera in pcDNA3.1 (Craig et al., 2004). Site-directed mutagenesis was performed using the QuikChange mutagenesis kit (Agilent Technologies) and verified by nucleotide sequencing (Source Bioscience). For consistency with previous studies, the numbering of amino acids in the α7 subunit mutated by site-directed mutagenesis is based on the predicted signal sequence cleavage site in the mature chick α7 subunit (Couturier et al., 1990).

### 2.4 Electrophysiological characterisation

Oocytes were isolated from adult female *Xenopus laevis* and defolliculated by treatment with type II collagenase (2.5 mg/ml; Gibco, ThermoFisher Scientific) in calcium-free Barth's solution containing 88 mM NaCl, 2.4 mM NaHCO_3_, 1 mM KCl, 0.82 mM MgSO_4_, and 15 mM Tris, pH 7.5, as described previously (Young et al., 2007). Heterologous expression was achieved by cytoplasmic injection of *in vitro* transcribed cRNA. Prior to *in vitro* synthesis of cRNA plasmid cDNA was linearized by restriction enzyme digestion and purified with QIAQuik PCR purification kit (Qiagen). *In vitro* synthesis of cRNA was performed using mMessage mMachine SP6 and T7 transcription kits (ThermoFisher Scientific). Oocytes were injected with approximately 9 ng cRNA using a Drummond variable volume microinjector. After injection, oocytes were incubated at 14 °C in a calcium-containing Barth's solution (composition, as above, but with 0.77 mM CaCl_2_) supplemented with antibiotics (100 units/ml penicillin, 100 μg/ml streptomycin, 4 μg/ml kanamycin, and 50 μg/ml tetracycline). Experiments were performed on oocytes after 3–5 days of incubation. Oocytes were placed in a recording chamber and continuously perfused with a modified Ringer's solution (115 mM NaCl, 2.5 mM KCl, 1.8 mM BaCl_2_, and 10 mM HEPES, pH 7.3) with a flow rate of approximately 15 ml/min. Two-electrode voltage-clamp recordings were performed using a Warner Instruments OC-725C amplifier (Havard Apparatus) with the oocyte membrane potential held at −60 mV, as described previously (Young et al., 2007; Gill et al., 2012). Application of compounds was controlled by LabChart software (AD Instruments) using a BPS-8 solenoid valve solution exchange system (ALA Scientific Inc). Allosteric modulators were pre-applied for 30 s before co-application with agonist.

### 2.5 Statistical analysis and curve fitting

Data are presented as means ± SEM of at least three independent experiments, that were conducted on separate oocytes. For multiple comparisons, statistical significance was determined with an unpaired one-way analysis of variance (ANOVA). Post-hoc analysis was performed with a Tukey multiple comparison test. For individual comparisons, statistical significance was determined using unpaired *t*-tests. In order to produce concentration-response curves for wild-type and mutant α7 receptors, current response data was normalised and fitted to the sigmoidal function:

$$I/I_{max}=\frac{1}{\left[1+10^{\left(\text{log}\left(\frac{EC_{50}}{\left[agonist\right]}\right)n_{H}\right)}\right]}$$

where I is the current and I_max_ is the maximum current. EC_50_ is the concentration of agonist that evokes 50% of the maximum current and n_H_ is the Hill coefficient. Inhibition curves were fitted using the following equation, where IC_50_ is the concentration of the antagonist that is required to inhibit the maximum response by 50%:

$$I/I_{max}=\frac{1}{\left[1+10^{\left(\text{log}\left(\frac{\left[antagonist\right]}{IC_{50}}\right)n_{H}\right)}\right]}$$

## 3 Results

### 3.1 Virtual screening

Virtual screening of the DrugBank database (Wishart et al., 2006) was performed with the aim of identifying novel allosteric modulators of the human α7 nAChR. Six pharmacophore queries (Fig. 1) were generated, based on the predicted bound conformation within the open and closed structural models of the α7 nAChR (Newcombe et al., 2017) of a series of 25 compounds that are known to have PAM activity on the α7 nAChR (see the representative ‘TQS-family’ structure illustrated in Fig. 2 and also the compounds identified as ‘TQS-family’ in the supplemental Table 1 of Newcombe et al., 2017). Validation runs, in which the pharmacophore queries were screened against 42 known active α7 nAChR PAMs, suggested that they were of high quality, as indicated by AUC values from ROC plots of 0.949 or greater for all queries (Table S2). For both the closed and open queries generated, query 2 had the best validation scores, with the highest AUC (0.971 and 0.967 respectively) as well as the highest early enrichment across 0.5%, 1% and 2% thresholds (Table S2 and Fig. S1). The DrugBank database (Wishart et al., 2006) was screened with the selected pharmacophore queries, after applying two filters (see Materials and Methods) to the top 100 hits from each query, 81 compounds were identified (Table S3).

### 3.2 Selection of compounds for functional characterisation

From the 81 compounds identified by virtual screening of the DrugBank database, four were selected for detailed functional characterisation on α7 nAChRs expressed in *Xenopus* oocytes (DB00487 [pefloxacin], DB00695 [furosemide], DB04763 and DB08122; Fig. 2). These were the highest-ranked members of the four classes of compounds that were represented in the 25 top-ranked virtual screening hits (Table S3). DB04763, a CAII inhibitor (Scozzafava et al., 2000), is the top-ranked compound identified by virtual screening. It is one of 22 CAII inhibitors in the 25 top-ranked compounds and also one of 36 such compounds in the 81 total hits (Table S3). DB08122 is the 3rd-ranked compound identified by virtual screening and is a CDK2 inhibitor (Bramson et al., 2001). It is the only example of a CDK2 inhibitor in the 25 top-ranked hits but it is one of seven CDK2 inhibitors within the 81 total hits (Table S3). Furosemide (DB00695) is the 6th-ranked hit from virtual screening and is a diuretic that acts on the Na^+^-K^+^-Cl^-^ cotransporter. No other compounds with a similar known mechanism of action were identified amongst the 81 virtual screening hits. Pefloxacin (DB00487), a fluroquinolone antibiotic (Wolfson and Hooper, 1989), is the 22nd-ranked hit and is the only example of this type of compound in the 25 top-ranked hits but it is one of 13 such compounds within the 81 total hits (Table S3). Overlays of the selected compounds with the original pharmacophore queries demonstrates their close match to the shape of the ‘TQS-family’ of PAMs (Fig. S2).

### 3.3 Functional characterisation

**Fig. 3:**
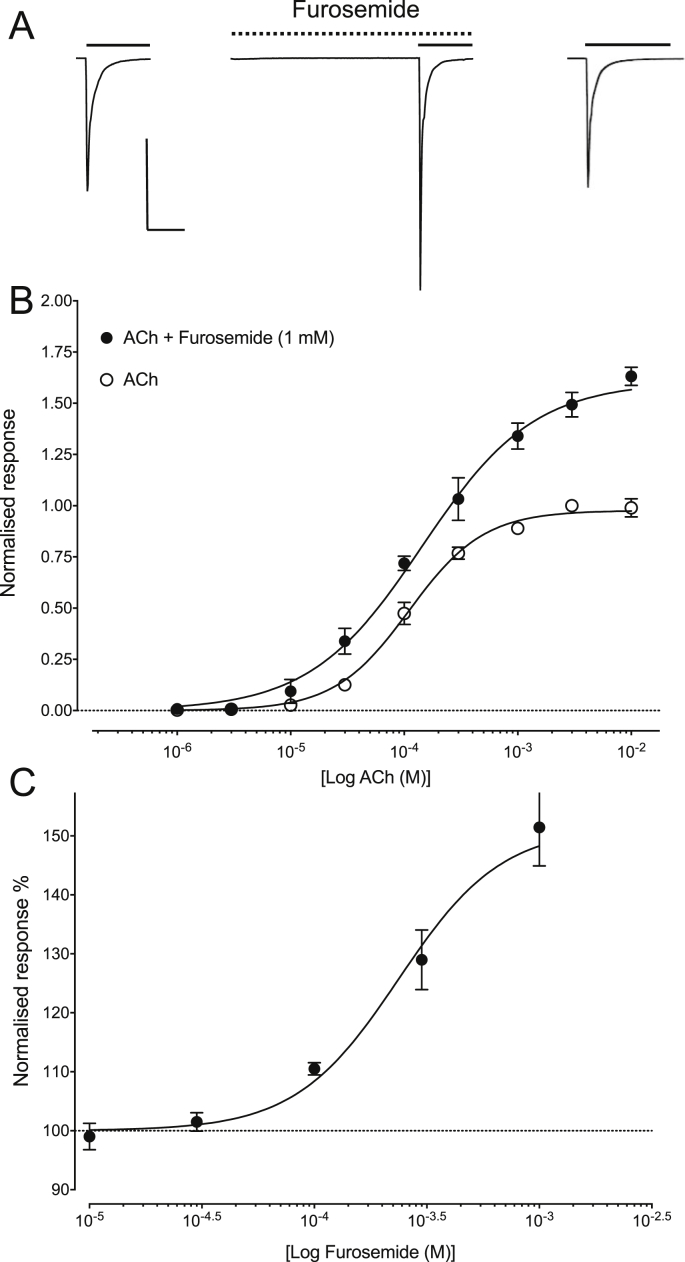
Functional characterisation of furosemide on the α7 nAChR. A) Representative traces illustrating responses of α7 nAChRs to ACh (100 μM; left), together with ACh responses from the same oocyte after pre- and co-application of furosemide (1 mM; middle). Also shown (right) is a response to ACh (100 μM) following wash. The vertical scale bar corresponds to 200 nA and the horizontal scale bars to 2.5 s. B) Responses with varying concentrations of ACh in the absence (open circles) and presence (closed circles) of a fixed concentration (1 mM) of furosemide. Data are normalised to the maximum ACh response and are means ± SEM of three independent experiments. C) Concentration-response data illustrating potentiation of responses to ACh (50 μM) by varying concentrations of furosemide. Data are normalised to the EC_20_ concentration of ACh (50 μM) and are means ± SEM of three independent experiments.

Selected compounds identified by virtual screening of the DrugBank database were tested on the human α7 nAChR expressed in *Xenopus* oocytes by two-electrode voltage-clamp recording. Initial studies focussed on the diuretic furosemide (ranked 6th; Table S3), which is the highest ranked compound identified by virtual screening that is available commercially. As has been reported previously (Couturier et al., 1990), application of ACh to α7 nAChRs resulted in rapidly desensitising inward currents (Fig. 3A). Furosemide (1 mM) had no effect on α7 nAChRs when applied alone but when tested under our standard protocol for PAMs (pre-application followed by co-application with ACh) this resulted in a dose-dependent potentiation of agonist-evoked responses (Fig. 3B). In contrast, in the absence of pre-application, no significant potentiation of ACh responses was observed (Fig. S3). When a range of concentrations of furosemide were co-applied with an EC_20_ concentration of ACh (50 μM), agonist-evoked responses were potentiated with an EC_50_ value of 0.2 ± 0.04 mM (n = 3) (Fig. 3C). At high concentrations of furosemide (1 mM), the maximum level of potentiation of ACh responses was 1.6 ± 0.1 fold (Fig. 3B), similar to that reported previously for type I PAMs that have chemical structures similar to the compounds used to generate the pharmacophore model (Gill-Thind et al., 2015).

**Fig. 4:**
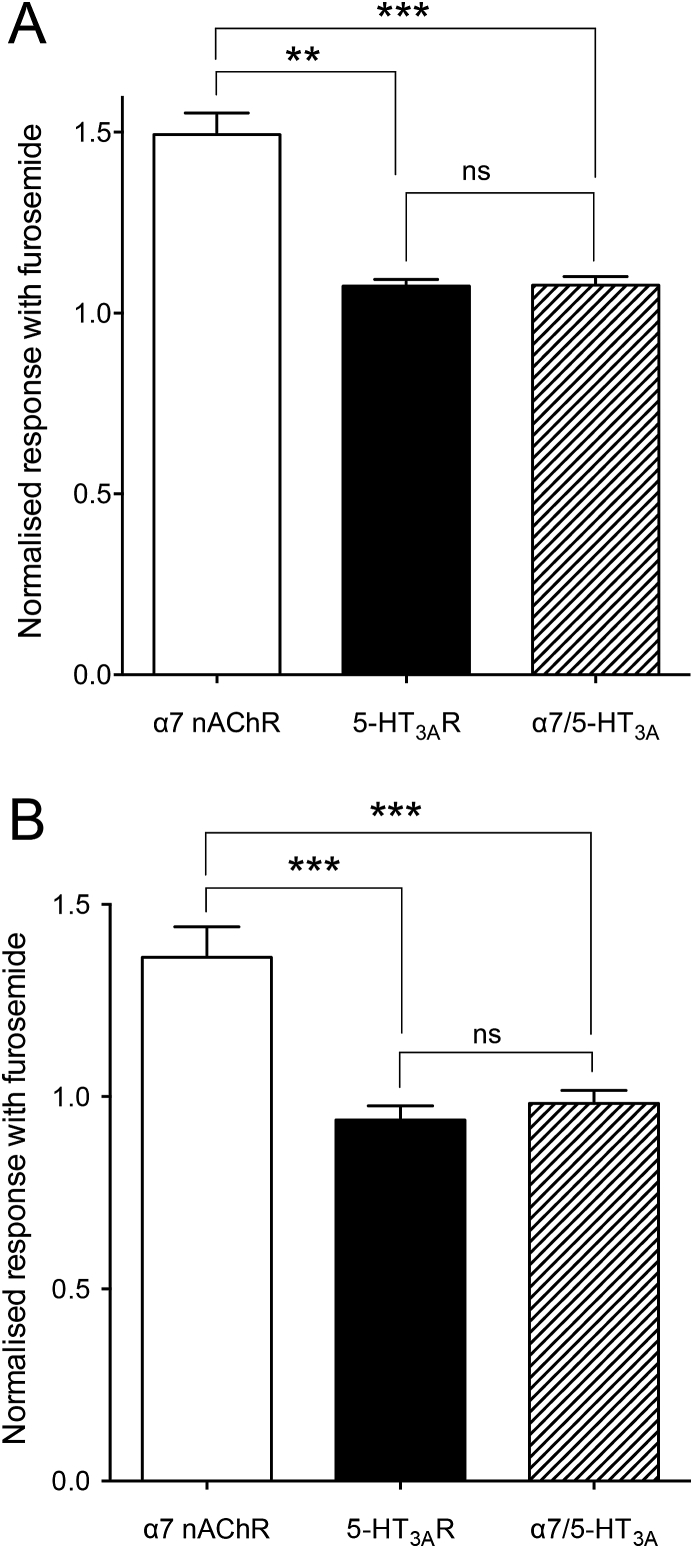
Functional characterisation of furosemide on α7 nAChR, 5-HT_3A_R and an α7/5-HT_3A_R chimera. The effect of furosemide was examined with either a maximal or an EC_50_ concentration of agonist (A and B, respectively). A) Furosemide (1 mM) was pre- and co-applied with a maximal concentration of agonist (3 mM ACh for α7 and α7/5-HT_3A_R chimera; 30 μM 5-HT for 5-HT_3A_R). B) Furosemide (1 mM) was pre and co-applied with an EC_50_ concentration of agonist (100 μM ACh for α7 and α7/5-HT_3A_R chimera; 1 μM 5-HT for 5-HT_3A_R). In both cases potentiation of the α7 nAChR was significantly greater than potentiation with the 5-HT_3A_R and with the α7/5-HT_3A_ chimera. Data are means ± SEM (n = 4–8). Significant differences are indicated (**P < 0.01, ***P < 0.001).

Furosemide was also tested on another closely related ligand-gated ion channel, the homomeric mouse 5-HT_3A_R (Maricq et al., 1991). As had been observed with the α7 nAChR, furosemide had no agonist effect when applied alone to the 5-HT_3A_R. When tested on maximal agonist concentrations (3 mM ACh for α7 and α7/5-HT_3A_R chimera; 30 μM 5-HT for 5-HT_3A_R), in contrast to the clear potentiating effect observed with furosemide on the human α7 nAChR, furosemide (1 mM) had only a very weak potentiating effect (108 ± 2%, n = 4, *P* = 0.027) on the mouse 5-HT_3A_R (Fig. 4). This is significantly less than the PAM effect observed in parallel experiments conducted with human α7 nAChR (149 ± 6%, n = 14; *P* = 0.002). If furosemide is acting at a transmembrane site, we would predict that it might have a similarly low potentiating effect on receptors generated by expression of a previously described chimera (α7/5-HT_3A_R) containing the extracellular domain of the human α7 nAChR fused to the transmembrane and C-terminal domain of the mouse 5-HT_3A_R (Craig et al., 2004). As predicted, furosemide exerted a very low PAM effect on the α7/5-HT_3A_R chimera (108 ± 2%) that was significantly less than its effect on α7 nAChR (*P* = 0.004) but was not significantly different from its effect on the 5-HT_3A_R (*P* = 0.963; Fig. 4). The experiment was also repeated with submaximal (EC_50_) agonist concentrations (100 μM ACh for α7 and α7/5-HT_3A_R chimera; 1 μM 5-HT for 5-HT_3A_R). As previously, in contrast to clear potentiation of α7 nAChRs (136 ± 7% n = 6), furosemide (1 mM) was observed to have no significant effect on 5-HT_3A_R (93.9 ± 3.7% n = 9, *P* = 0.137) or on the α7/5-HT_3A_R chimera (98.2 ± 3.4% n = 8, *P* = 0.619). Thus, studies conducted with maximal and submaximal agonist concentrations are consistent with furosemide potentiating the α7 nAChR by acting at a site other than the extracellular domain.

**Fig. 5:**
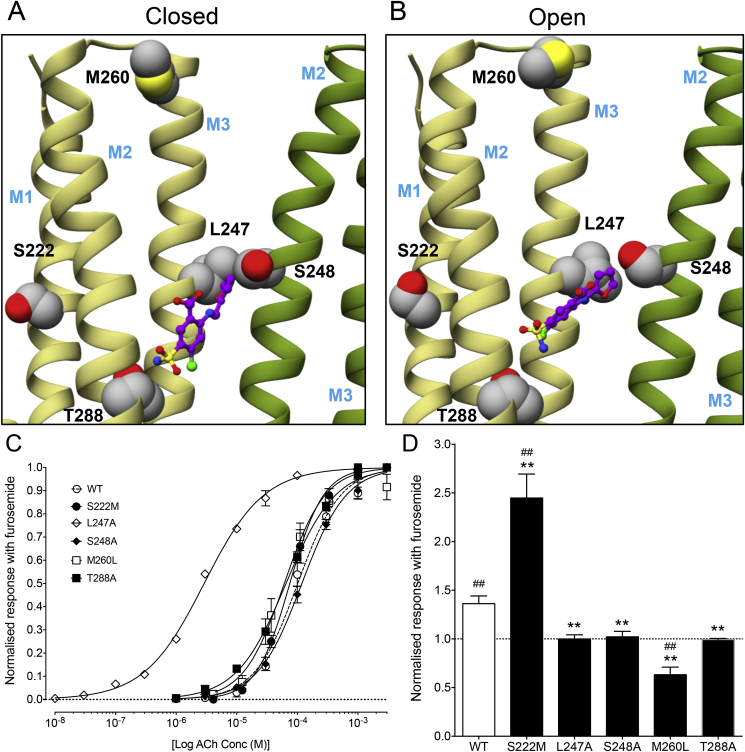
Influence of α7 nAChR mutations on the allosteric modulatory effect of furosemide. The docked position of furosemide is shown in the closed (A) and open (B) structural model of the α7 nAChR transmembrane region. The TM1-3 helices of the principal subunit (khaki) and TM2 and TM3 helices of the complimentary subunit (green) are shown. Amino acids examined by site-directed mutagenesis are indicated. C) ACh dose-response curves determined with wild-type α7 nAChR (dashed line) and with α7 nAChRs containing single point mutations S222M, L247A, S248A, M260L and T288A. Data are means of at least three independent experiments. D) Bar chart illustrating the influence of furosemide (1 mM) on responses to an EC_50_ concentration of ACh (10 μM for L247A and 100 μM for wild-type and all other mutated receptors). Data are normalised to the response observed in the same oocyte in the absence of furosemide. Data are means ± SEM of at least three independent experiments. Significant differences from wild-type are indicated (*P < 0.05, **P < 0.01). In addition, significant differences from agonist responses in the absence of furosemide are indicated (#*P* < 0.05, ##*P* < 0.01). (For interpretation of the references to color in this figure legend, the reader is referred to the Web version of this article.)

Given that the pharmacophores used for virtual screening were based on allosteric modulators that are predicted to bind in the α7 nAChR transmembrane region, the simplest explanation for the PAM effects observed with furosemide is that it acts at a similar transmembrane site. Previous studies have revealed that the effects of known α7 PAMs can be altered by single point mutations in the transmembrane domain (Young et al., 2008; Gill et al., 2011; Chatzidaki et al., 2015). Docking studies with furosemide into our revised structural model of the α7 nAChR (Newcombe et al., 2017) identified three amino acids in close proximity (within 3.5 Å) to the predicted furosemide binding site within the inter-subunit transmembrane region (L247, S248 and T288; Fig. 5), which are predicted to form hydrogen bonds (T288) or have hydrophobic interactions (L247 and S248) with the ligand. Each of these amino acids were individually mutated to alanine (L247A, S248A and T288A) and the effect of the mutations examined by expression of mutated α7 nAChRs in *Xenopus* oocytes. Two of the mutations (S248A and T288A) had no significant effect on sensitivity to the orthosteric agonist ACh (Fig. 5C). However, L247A caused a reduction in the rate of agonist-evoked desensitisation and a leftward shift in the dose-response curve for ACh (EC_50_ = 9.5 ± 0.4 μM, n = 3, *P* = 0.001; Fig. 5C), as has been reported previously for other mutations at this position (Revah et al., 1991). All three mutations (L247A, S248A and T288A) abolished potentiation by furosemide (1 mM) of agonist-evoked responses (Fig. 5D). The ACh-evoked responses in the presence of furosemide (1 mM) on α7 nAChRs containing the L247A, S248A and T288A mutations were not significantly different from control responses (99.9 ± 4.3% n = 4, *P* = 0.980; 102 ± 5.6% n = 6, *P* = 0.712; and 99.2 ± 1.2% n = 6, *P* = 0.568, respectively). These changes in response were significantly different in comparison to ACh-evoked responses on the wild-type α7 nAChR in the presence of furosemide (1 mM) (*P* = 0.012, 0.011 and 0.006, respectively). In addition, we examined two other α7 nAChR transmembrane mutations (S222M and M260L) that have been shown previously to influence allosteric modulation (Young et al., 2008; Chatzidaki et al., 2015; Newcombe et al., 2017). Neither mutation had a significant effect on sensitivity to ACh (Fig. 5C) but both altered sensitivity to furosemide (Fig. 5D). Mutation S222M caused a significant (*P* = 0.0018) increase in potentiation of an EC_50_ concentration of ACh by furosemide (1 mM) compared with wild-type receptor (potentiation of 244 ± 25%, n = 4, compared with 136 ± 7% for wild-type, n = 6; Fig. 5D). Mutation M260L converted furosemide into an antagonist. Agonist-evoked responses in the presence of furosemide (1 mM) were 63 ± 8% (n = 5) of the control response, in comparison to 136 ± 7% (n = 6) for the wild-type receptor (Fig. 5D).

**Fig. 6:**
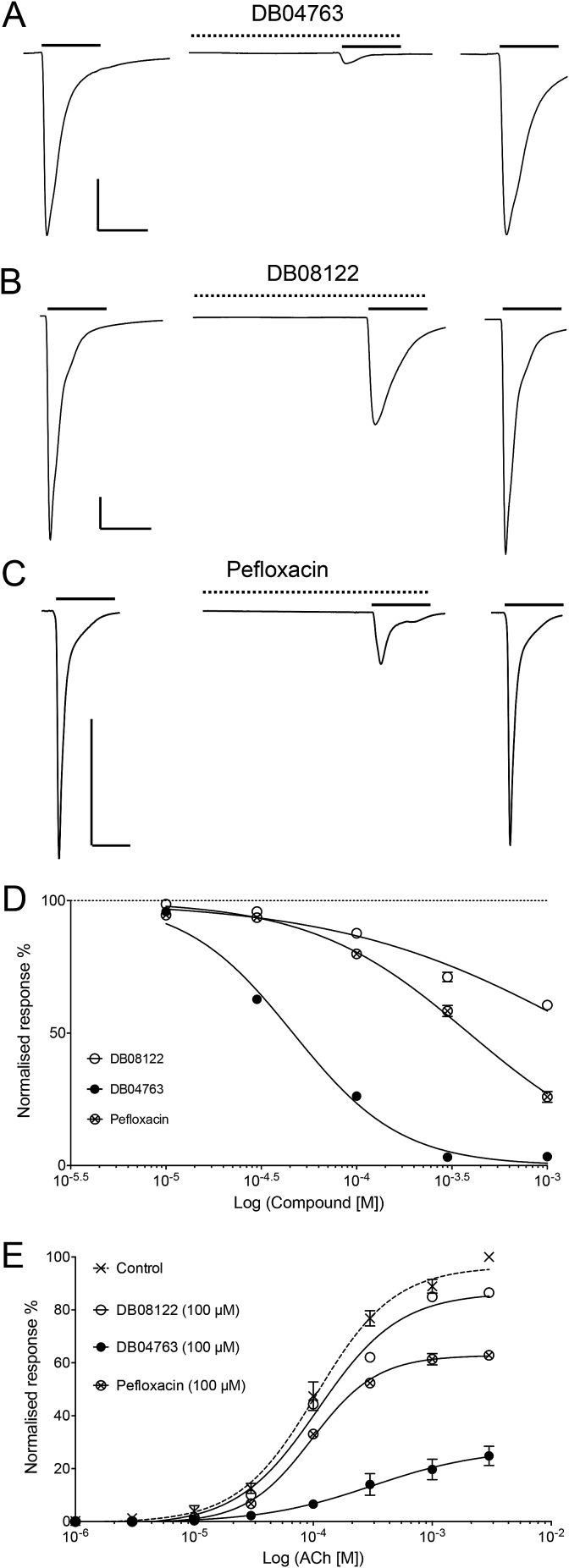
Functional characterisation of DB04763, DB08122 and pefloxacin on the α7 nAChR. A-C) Representative traces, from oocytes expressing the α7 nAChR, in response to ACh (100 μM; left), together with an ACh response from the same oocyte after pre- and co-application of test compound (1 mM; middle). Also shown are responses to ACh (100 μM) after a 2 min wash (right). Data are shown for DB04763 (A), DB08122 (B) and pefloxacin (C), all at 1 mM. Vertical scale bars correspond to 500 nA and horizontal scale bars correspond to 5 s. D) Concentration-response data illustrating antagonism of responses to ACh (100 μM) by varying concentrations of DB04763 (filled circles), DB08122 (open circles) and pefloxacin (crossed circles). Data are normalised to the response to an EC_50_ concentration of ACh (100 μM) and are means ± SEM of three independent experiments. E) Responses to varying concentrations of ACh in the presence of a fixed concentration (100 μM) of DB04763 (filled circles), DB08122 (open circles) and pefloxacin (crossed circles). Data are normalised to the response to a maximum concentration of ACh (3 mM) and are means ± SEM of 3–4 independent experiments.

Three other compounds identified by virtual screening of the DrugBank database were also tested on α7 nAChRs expressed in *Xenopus* oocytes (DB04763, DB08122 and pefloxacin), corresponding to the top ranked CAII inhibitor, CDK2 inhibitor and fluoroquinolone antibiotic hits, respectively. None of the three compounds had any significant effect when applied alone to α7 nAChRs but all significantly inhibited agonist-evoked responses in a dose-dependent manner when co-applied with ACh (Fig. 6A–D). Note, although a standard protocol was used for all of the data that is presented (whereby compounds were pre-applied for 30s, followed by co-application), in contrast to the modulatory effect observed with furosemide which required pre-application, significant antagonism was observed with all three compounds in the absence of pre-application (Fig. S3). At high concentrations (1 mM) DB04763 caused an almost complete block of responses to an EC_50_ concentration of ACh (3.4 ± 0.9% of the control response, n = 3, *P* = 0.0001). In contrast, high concentrations (1 mM) of DB08122 and pefloxacin caused a partial block of responses to an EC_50_ concentration of ACh (60.6 ± 1.2%, n = 3, *P* = 0.0001 and 25.9 ± 2.1%, n = 5, *P* = 0.0001, of the control response respectively) (Fig. 6D). This level of antagonism is similar to that reported previously for NAMs that have chemical structures similar to the compounds used to generate the pharmacophore model (Gill-Thind et al., 2015). When a range of concentrations of DB04763, DB08122 and pefloxacin were co-applied with an EC_50_ concentration of ACh (100 μM), agonist-evoked responses were inhibited with IC_50_ values of 46.4 ± 2.2 μM (n = 3),1.7 ± 0.3 mM (n = 3) and 388 ± 2.1 μM (n = 5), respectively (Fig. 6D). When each of the three compounds (at 100 μM) was co-applied with a range of ACh concentrations, the antagonism was found to be non-surmountable (Fig. 6E), which suggests a non-competitive mechanism of action and is consistent with the compounds acting as NAMs.

**Fig. 7:**
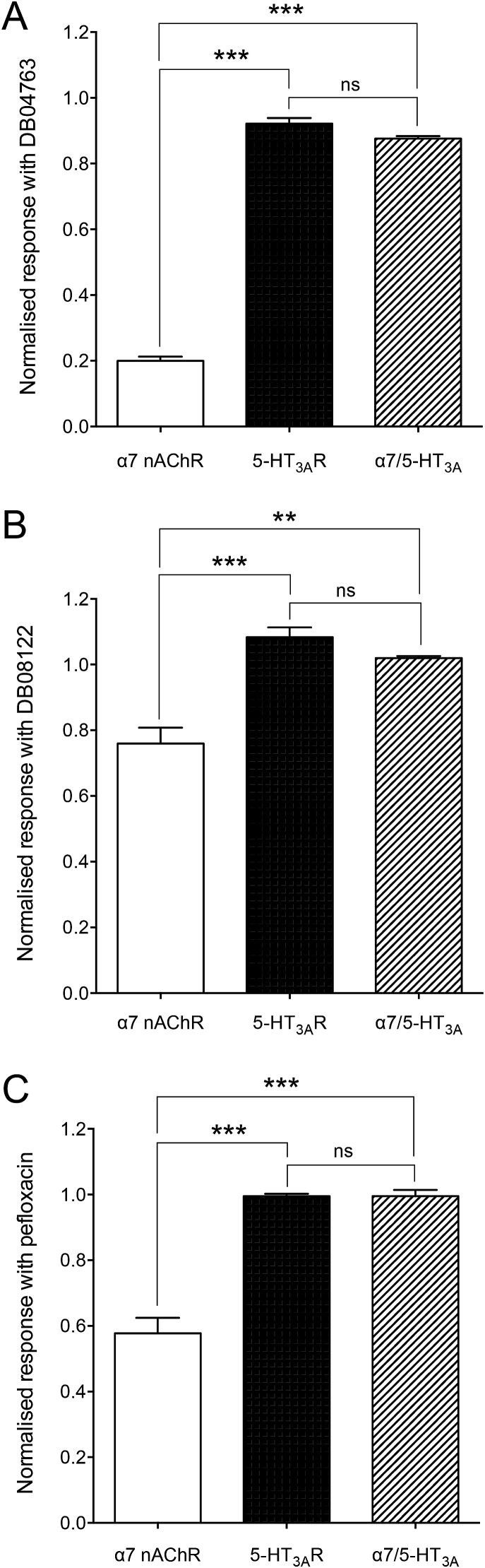
Functional characterisation of DB04763, DB08122 and pefloxacin on the 5-HT_3A_R and α7/5-HT_3A_R chimera. The effects of DB04763 (A), DB08122 (B) and pefloxacin (C), were examined by pre- and co-application (100 μM) with a maximal concentration of agonist (3 mM ACh for α7 and α7/5-HT_3A_R chimera; 30 μM 5-HT for 5-HT_3A_R) on the α7 nAChR, 5-HT_3A_R and α7/5-HT_3A_R chimera. All three of the compounds inhibited the 5-HT_3A_R and α7/5-HT_3A_R chimera to a significantly lower extent than was observed with the α7 nAChR. Data are means ± SEM (n = 4–6). Significant differences are indicated (**P < 0.01, ***P < 0.001).

As described above for furosemide, the influence of DB04763, DB08122 and pefloxacin (all at 100 μM) were examined on responses evoked by a maximal agonist concentration on α7 nAChRs, the 5-HT_3A_Rs and the α7/5-HT_3A_R chimera (Fig. 7). DB04763 inhibited responses on α7 nAChRs to 20.0 ± 1.3% (n = 6), DB08122 to 76.0 ± 4.8% (n = 6) and pefloxacin to 57.7 ± 4.7% (n = 6). In contrast DB08122 and pefloxacin did not cause significant inhibition on responses on 5-HT_3A_Rs and responses were inhibited to only a small extent by DB04763 (92.1 ± 1.7%, n = 6, *P* = 0.038). When tested on the α7/5-HT_3A_R chimera the effects of all three compounds were significantly different to that observed on α7 nAChRs (DB04763, *P* < 0.0001; DB08122, *P* = 0.0012 and pefloxacin, *P* < 0.0001) and not significantly different to their effects on 5-HT_3A_Rs (Fig. 7). These findings are consistent with these antagonists acting at a site other than the extracellular domain. These experiments were also repeated to examine the effects of the compounds on responses to a submaximal (EC_50_) concentration of agonist. For all compounds tested, higher levels of inhibition were observed with 5-HT_3A_Rs than had been the case with maximal agonist concentrations. Because relatively high levels of inhibition were observed on both α7 nAChRs and on 5-HT_3A_Rs, it was felt that studies with the α7/5-HT_3A_R chimera would not be helpful in identifying the receptor domain through which the antagonist effect was occurring. However the data obtained with maximal concentrations of agonist are consistent with the three compounds exerting an inhibitory effect on α7 nAChRs via a transmembrane site.

**Fig. 8:**
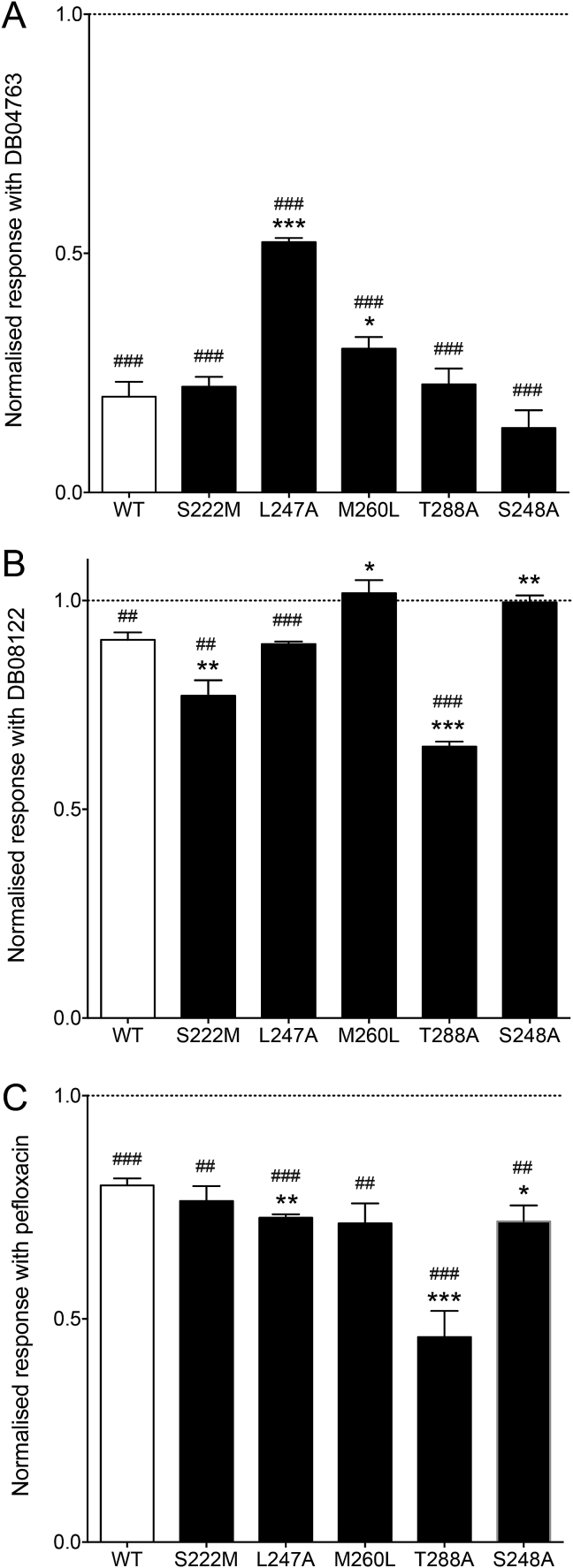
Influence of α7 nAChR mutations on the allosteric modulatory effect of DB04763, DB08122 and pefloxacin. Data are shown for DB04763 (A), DB08122 (B) and pefloxacin (C). Bar charts illustrate the influence of the compounds (100 μM) on responses to an EC_50_ concentration of ACh (10 μM for L247A and 100 μM for wild-type and all other mutated receptors). Data are normalised to the response observed in the same oocyte in the absence of the compounds. Data are means ± SEM of at least three independent experiments. Significant differences from wild-type are indicated (*P < 0.05, **P < 0.01, ***P < 0.001). In addition, significant differences from agonist responses in the absence of the compounds are indicated (##*P < *0.01, ###*P < *0.001).

In addition, the three antagonists (DB04763, DB08122 and pefloxacin) were examined on α7 nAChRs containing the series of single transmembrane mutations that had been examined with furosemide (S222M, L247A, S248A M260L and T288A) (Fig. 8). The effect of the compounds (100 μM) was examined on responses to a submaximal (EC_50_) concentration of ACh. In comparison to the effects of the compounds on wild-type receptors, significant differences in the levels of antagonism were observed with DB04763 on two of the mutated receptors (L247A and M260L), with DB08122 on four of the mutated receptors (S222M, M260L, T288A and S248A) and with pefloxacin on three of the mutated receptors (L247A, T288A and S248A) (Fig. 8). In comparison to normalised responses observed on wild-type receptors in the presence of DB04763 (20.1 ± 3.1%, n = 6), responses were significantly larger on L247A (52.4 ± 0.8% n = 6, *P* < 0.0001) and on M260L (30.1 ± 2.4% n = 8, *P* = 0.0243). In comparison to normalised responses observed on wild-type receptors in the presence of DB08122 (90.6 ± 1.8%, n = 6), responses were significantly reduced on S222M (77.2 ± 3.7% n = 6, *P* = 0.0087) and on T288A (65.0 ± 1.2% n = 6, *P* < 0.0001). In contrast, antagonism by DB08122 was abolished on M260L (101 ± 3.1% n = 6, *P* = 0.0112) and on S248A (99.6 ± 1.6% n = 6, *P* = 0.039). In comparison to normalised responses observed on wild-type receptors in the presence of pefloxacin (79.9 ± 1.6%, n = 7), responses were significantly smaller on L247A (72.7 ± 0.7%, n = 6, *P* = 0.0025), S248A (71.8 ± 3.5%, n = 5, *P* = 0.0458) and T288A (45.9 ± 5.8%, n = 7, *P* = 0.0001) (Fig. 8).

## 4 Discussion

The success of our virtual screening approach, as illustrated by evidence that all four of the compounds tested in functional assays displayed allosteric modulatory activity on α7 nAChRs, provides further support for our revised structural models of the α7 nAChR (Newcombe et al., 2017). The revised α7 models were based upon the electron diffraction structures of the *Torpedo* nAChR (Unwin, 2005; Unwin and Fujiyoshi, 2012) after correcting what had been identified previously as an error in the assignment of amino acids within the transmembrane region of the *Torpedo* nAChR structure (Corringer et al., 2010; Hibbs and Gouaux, 2011; Mnatsakanyan and Jansen, 2013). As discussed previously (Newcombe et al., 2017), an advantage of using a corrected *Torpedo* nAChR structure (derived from receptors embedded in a lipid membrane), rather than any of the more recently determined structures of detergent-solubilised receptors would avoid concerns that the removal of membrane lipids during protein purification might influence the overall transmembrane structure. In addition, it avoided the possibility that the close packing of receptors in three-dimensional crystals might alter protein conformation. A further advantage of using the corrected *Torpedo* nAChR structures as a starting point for generating α7 structural models is that *Torpedo* nAChR structures are available that correspond to both an open and closed state (Unwin, 2005; Unwin and Fujiyoshi, 2012). Although PAMs can be viewed as stabilizing the open conformation of the receptor (and NAMs the closed conformation), our initial docking studies were performed with both open and closed conformations to avoid a bias and this may have accounted for our identification by virtual screening of both PAMs and NAMs.

Several previous reports of virtual screening aimed at the identification of nicotinic ligands have been based on structural models of nAChR extracellular domains (Peng et al., 2010; Mahasenan et al., 2011; Chen et al., 2013; Kombo and Bencherif, 2013; Zheng et al., 2016; Leffler et al., 2017) or on the structure of the acetylcholine binding protein, which is homologous to the nAChR extracellular domain (Babakhani et al., 2009; Ulens et al., 2009; Utsintong et al., 2009, 2012). In contrast, our aim was to perform virtual screening with pharmacophores focusing on the transmembrane domain of the human α7 nAChRs and our recent revised models (Newcombe et al., 2017). Remarkably, all four of the compounds that we have examined in functional assays exhibit allosteric modulatory effects when tested on expressed α7 nAChRs. These compounds represent four functional classes (CAII inhibitors, CDK2 inhibitors, diuretics targeting the Na^+^-K^+^-Cl^-^ cotransporter and fluoroquinolone antibiotics) and were selected because they represent all compound classes within the top 25 virtual screening hits and are also representative of 58 of the total 81 compounds identified.

The highest ranked hit from virtual screening that was available commercially for purchase was furosemide (the 6th-ranked hit; Table S3), a loop diuretic that acts on the Na^+^-K^+^-2Cl^-^ cotransporter (O'Grady et al., 1987). Interestingly, in a study conducted almost 30 years ago it was reported that furosemide increased contraction of rat urinary bladder by potentiating the action of acetylcholine, and that this effect could be blocked by the nicotinic receptor antagonist hexamethonium (Okpukpara and Akah, 1989). In addition, there is more recent evidence indicating that functional α7 nAChRs are expressed in rat urinary bladder epithelial cells (Beckel et al., 2006). Consequently, our finding that furosemide acts as a PAM of α7 nAChRs provides a plausible explanation for the previously reported effects of furosemide in potentiating acetylcholine-induced bladder contraction (Okpukpara and Akah, 1989). Previous studies have also demonstrated that furosemide acts as an antagonist of GABA_A_Rs (Korpi et al., 1995), which are also members of the superfamily of pentameric ligand-gated ion channels. It has also been suggested that furosemide interacts with GABA_A_R via a transmembrane binding site (Thompson et al., 1999), a conclusion that is consistent with the findings in the present study concerning the α7 nAChR. Furosemide potentiated agonist responses in α7 nAChRs but did not alter the rate of agonist-induced desensitisation (Fig. 3B), indicating that furosemide can be considered a type I PAM (Bertrand and Gopalakrishnan, 2007).

As described above, the pharmacophore queries used for virtual screening (Newcombe et al., 2017) were based on PAMs that are predicted to bind in the α7 nAChR transmembrane domain (Gill et al., 2011, 2012; Chatzidaki et al., 2015; Gill-Thind et al., 2015). This suggests that furosemide may exert its PAM effects by binding to a similar transmembrane site. Evidence supporting this conclusion has been provided by studies conducted with 5-HT_3A_R and an α7/5-HT_3A_R chimera containing the extracellular domain of the α7 subunit fused to the transmembrane and C-terminal domain of the 5-HT_3A_R. Furosemide displayed a significantly lower level of potentiation on both the 5-HT_3A_R and on the chimera (Fig. 4). Results of docking simulations of furosemide with our revised α7 structural models are also consistent with furosemide binding to an inter-subunit transmembrane site (Fig. 5). On the basis of these docking studies, three transmembrane amino acids (L247, S248 and T288) were identified as being in close proximity to the predicted binding site of furosemide (Fig. 5A–B). Point mutations introduced at all three of these positions *abolished* potentiation by furosemide (Fig. 5D), which is consistent with the possibility of altering the interaction of furosemide with a transmembrane binding site. The effects of furosemide were also altered by two additional transmembrane mutations (S222M and M260L), both of which have been reported previously to alter the properties of PAMs that are thought to interact with the transmembrane region (Young et al., 2008; Chatzidaki et al., 2015; Newcombe et al., 2017). Although these two transmembrane mutations are located further from the predicted transmembrane allosteric binding site, it seems reasonable to think that a transmembrane mutation may exert effects on allosteric modulators through a longer-range alteration in transmembrane structure or alter an allosteric gating mechanism.

The other three compounds examined (DB04763, DB08122 and pefloxacin) acted as antagonists of α7 nAChR (Fig. 6). In all three cases the antagonism was non-surmountable (Fig. 6E), which indicates a non-competitive mechanism of action. This is consistent with the conclusion that they are acting as α7 nAChR NAMs. As was the case with furosemide, the extent of modulation observed with the three NAMs was altered significantly by multiple transmembrane mutations (Fig. 8). Similarly, as with furosemide, evidence from studies conducted with an α7/5-HT_3A_R chimera are consistent with the three antagonists acting via a transmembrane binding site. In all cases antagonism was either abolished or significantly reduced on the α7/5-HT_3A_R chimera (Fig. 7). Previous studies have demonstrated that even very small changes in compound structure, for example changes in the extent of methyl-substitution of a single aromatic ring, can convert α7 nAChR PAMs into NAMs (Gill-Thind et al., 2015). Therefore, it is not unexpected that virtual screening conducted with pharmacophore queries derived from α7 nAChR PAMs would identify compounds acting as NAMs.

We are unaware of any previous evidence indicating that inhibitors of CAII (such as DB04763) or of CDK2 (such as DB08122) can exert allosteric modulatory effects on nAChRs. Pefloxacin is a member of the quinolone class of broad-spectrum antibiotics that interfere with bacterial DNA replication via action on DNA gyrase (Aldred et al., 2014). Interestingly, previous studies have indicated that quinolone antibiotics can act as antagonists of GABA_A_Rs and it has been suggested that this may explain some of the adverse CNS side effects associated with quinolone antibiotics (Halliwell et al., 1993).

For some of the compounds examined (furosemide and DB04763) pre-application prior to co-application with agonist was required to see maximal modulatory effects (Fig. S3). It is possible that this may reflect restricted access and slower equilibration at a transmembrane binding site compared to agonist binding to the extracellular orthosteric site. Indeed a slower rate of activation is observed for allosteric agonists that are thought to interact with a transmembrane binding site compared to orthosteric agonists (Gill et al., 2011, 2012; Chatzidaki et al., 2015; Gill-Thind et al., 2015).

In summary, we have performed virtual screening of the DrugBank database with pharmacophore models that were generated from the structures of PAMs that have been shown previously to interact with an inter-subunit transmembrane site of the α7 nAChR. Four compounds, representative of the highest-ranking hits from the virtual screen, were tested experimentally by electrophysiology and mutagenesis studies and all were found to display properties on α7 nAChR consistent with them acting as allosteric modulators.
