# Supplementary material for: Identification by virtual screening and functional characterisation of novel positive and negative allosteric modulators of the α7 nicotinic acetylcholine receptor
Source: Neuropharmacology. 2018 Sep 1;139:194–204. doi: 10.1016/j.neuropharm.2018.07.009 (PMC6078708; doi:10.1016/j.neuropharm.2018.07.009)
Supplement: Multimedia component 1 [file mmc1.pdf]

Supplementary data

Identification by virtual screening and functional characterisation of novel positive and negative allosteric modulators of the α7 nicotinic acetylcholine receptor

Charles L.C. Smelt, Victoria R. Sanders, Joseph Newcombe, Richard P. Burt, Tom D. Sheppard, Maya Topf and Neil S. Millar

Supplementary Methods

Synthesis of 2,4,6-trimethyl-1-(4-sulfamoylphenethyl)pyridin-1-ium tetrafluoroborate (DB04763)

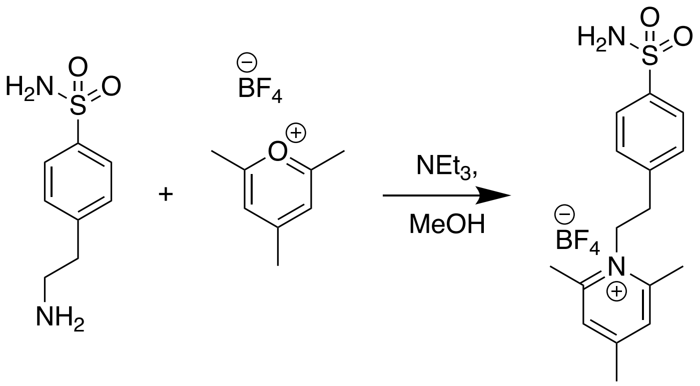

Triethylamine (350 µL, 253 mg, 2.5 mmol) was added to a solution of 2,4,6-trimethylpyrylium tetrafluoroborate (525 mg, 2.5 mmol) and 4-(2-aminoethyl)benzenesulfonamide (500 mg, 2.5 mmol) in methanol (15 mL), and the mixture heated at reflux for 15 min. Glacial acetic acid (286 µL, 5 mmol) was added, and the mixture stirred at reflux for a further 60 min. The reaction mixture was cooled to 4 °C and left to stand overnight in a fridge. Diethyl ether (100 mL) was added to the cooled reaction mixture and the precipitate collected by filtration. The precipitate was treated with concentrated ammonia solution, reprecipitated with perchloric acid and recrystallized from a solution of water containing 2% hypochlorous acid to afford the tetrafluoroborate salt of the title compound as a crystalline white solid (468 mg, 1.19 mmol, 48%).

Mp 169-171 °C; ^{1}H NMR (600 MHz, *methanol-d$_4$*) δ_H 7.88 (d, *J* = 8.3 Hz, 2H), 7.68 (s, 2H), 7.46 (d, *J* = 8.3 Hz, 2H), 4.77-4.71 (m, 2H), 3.30-3.27 (m, 2H), 2.80 (s, 6H), 2.56 (s, 3H); ^{13}C NMR (151 MHz, *methanol-d$_4$*) δ_C 160.0, 156.1, 144.5, 142.0, 130.7, 129.9, 127.9, 53.8, 34.7, 21.4, 21.1; ^{19}F NMR (282 MHz, *DMSO-d$_6$*) δ_F -148.19 (br s, $[^{10}BF_4]^-$), -148.25 (br s, $[^{11}BF_4]^-$); ^{11}B NMR (225 MHz, *DMSO-d$_6$*) δ_B -1; HRMS (ESI): Found: $[M]^+$ 305.1321 $C_{16}H_{21}N_2O_2S$ requires 305.1324.

Synthesis of (*Z*)-*N*-methyl-4-(((2-oxoindolin-3-ylidene)methyl)amino)benzenesulfonamide (DB08122)

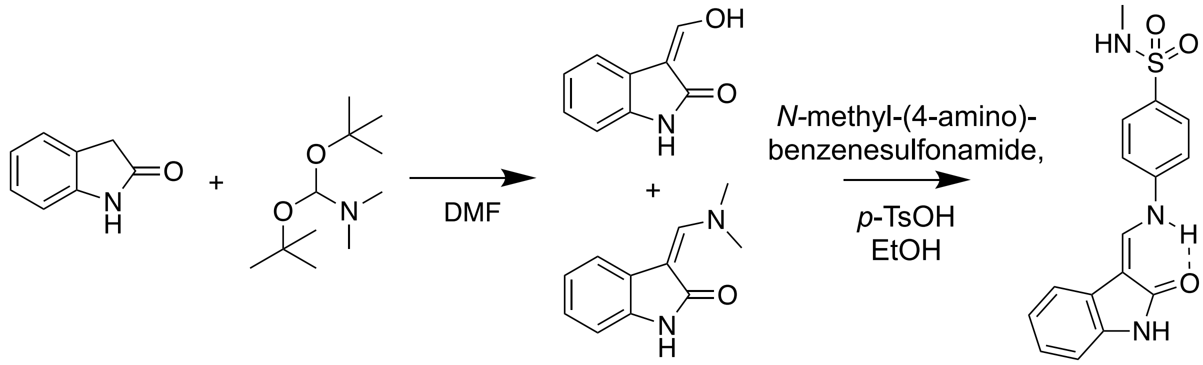

N,N-Dimethylformamide di-(*tert*-butyl)-acetal (1.15 mL, 975 mg, 4.8 mmol) was added to a solution of oxindole (500 mg, 3.75 mmol) in *N,N*-dimethylformamide (6.0 mL). The mixture was stirred at room temperature for 2 h. The crude mixture was concentrated *in vacuo* and the residue left overnight at room temperature. The crystalline solid and mother liquor were diluted with diethyl ether (70 mL) and the precipitate removed by filtration. The filtrate was washed with diethyl ether (2 x 20 mL) to give a golden crystalline solid which was found to be a 1:0.4 ratio (NMR) of 3-((dimethylamino)methylene)indolin-2-one with 3-(hydroxymethylene)indolin-2-one (334 mg), which was used in the next reaction without further purification.

A mixture of 3-((dimethylamino)methylene)indolin-2-one and 3-(hydroxymethylene)indolin-2-one (282 mg, ~1.55 mmol, 1.35 mmol 3-((dimethylamino)methylene)indolin-2-one), *N*-methyl-(4-amino)-benzenesulfonamide (251 mg, 1.35 mmol) and *p*-toluenesulfonic acid monohydrate (256 mg, 1.35 mmol) in ethanol (14 mL) was heated at reflux overnight. The reaction mixture was cooled and the precipitate removed by filtration and washed with cold ethanol to afford the desired product as a yellow crystalline solid (277 mg, 0.84 mmol, 22% over 2 steps).

Mp 234-236 °C; ^{1}H NMR (700 MHz, *DMSO-d$_6$*) δ_H 10.84 (d, *J* = 12.2 Hz, 1H), 10.56 (s, 1H), 6.62 (d, *J* = 12.2 Hz, 1H), 7.72 (d, *J* = 8.7 Hz, 2H), 7.60 (d, *J* = 7.5 Hz, 1H), 7.57 (d, *J* = 8.7 Hz, 2H), 7.32 (q, *J* = 5.1 Hz, 1H), 7.04 (t, *J* = 7.6 Hz, 1H), 6.94 (t, *J* = 7.5 Hz, 1H), 6.85 (d, *J* = 7.6 Hz, 1H), 2.40 (d, *J* = 5.1 Hz, 3H); ^{13}C NMR (176 MHz, DMSO-*d$_6$*) δ_C 169.8, 143.4, 137.5, 136.6, 132.5, 128.5, 124.8, 123.8, 120.6, 117.6, 115.7, 109.3, 101.8, 28.6; HRMS (ESI): Found: $[M+H]^+$ 330.0907 $C_{16}H_{16}N_3O_3S$ requires 330.0907.

Table S1. Previously identified α7 nAChR type II PAMs

Structure	Name	Reference
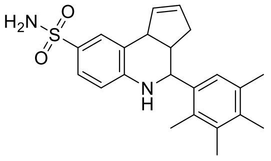	4-(2,3,4,5-tetramethylphenyl)-3a,4,5,9b-tetrahydro-3H-cyclopenta[c]quinoline-8-sulfonamide	(1)
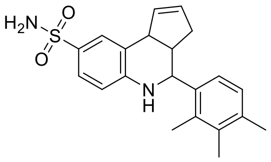	4-(2,3,4-trimethylphenyl)-3a,4,5,9b-tetrahydro-3H-cyclopenta[c]quinoline-8-sulfonamide	(1)
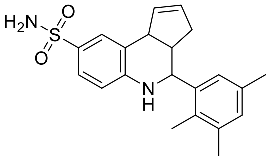	4-(2,3,5-trimethylphenyl)-3a,4,5,9b-tetrahydro-3H-cyclopenta[c]quinoline-8-sulfonamide	(1)
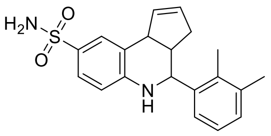	4-(2,3-dimethylphenyl)-3a,4,5,9b-tetrahydro-3H-cyclopenta[c]quinoline-8-sulfonamide	(1)
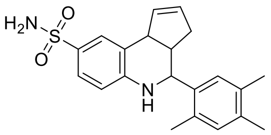	4-(2,4,5-trimethylphenyl)-3a,4,5,9b-tetrahydro-3H-cyclopenta[c]quinoline-8-sulfonamide	(1)
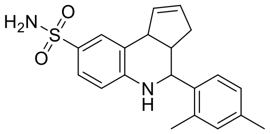	4-(2,4-dimethylphenyl)-3a,4,5,9b-tetrahydro-3H-cyclopenta[c]quinoline-8-sulfonamide	(1)
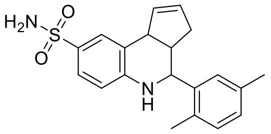	4-(2,5-dimethylphenyl)-3a,4,5,9b-tetrahydro-3H-cyclopenta[c]quinoline-8-sulfonamide	(1)
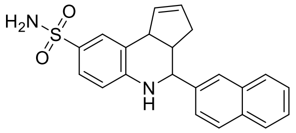	4-(naphthalen-2-yl)-3a,4,5,9b-tetrahydro-3H-cyclopenta[c]quinoline-8-sulfonamide	(2)

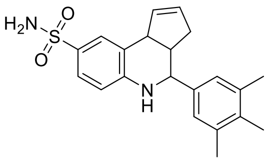	4-(3,4,5-trimethylphenyl)-3a,4,5,9b-tetrahydro-3H-cyclopenta[c]quinoline-8-sulfonamide	(1)
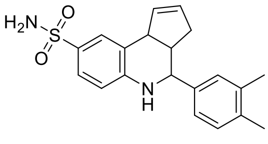	4-(3,4-dimethylphenyl)-3a,4,5,9b-tetrahydro-3H-cyclopenta[c]quinoline-8-sulfonamide	(1)
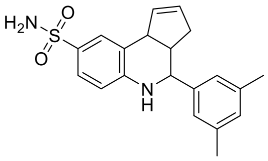	4-(3,5-dimethylphenyl)-3a,4,5,9b-tetrahydro-3H-cyclopenta[c]quinoline-8-sulfonamide	(1)
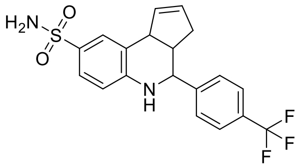	4-(4-(trifluoromethyl)phenyl)-3a,4,5,9b-tetrahydro-3H-cyclopenta[c]quinoline-8-sulfonamide	(2)
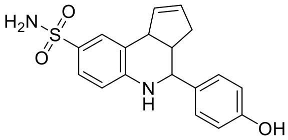	4-(4-hydroxyphenyl)-3a,4,5,9b-tetrahydro-3H-cyclopenta[c]quinoline-8-sulfonamide	(2)
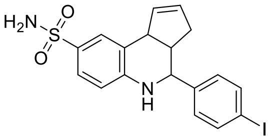	4-(4-iodophenyl)-3a,4,5,9b-tetrahydro-3H-cyclopenta[c]quinoline-8-sulfonamide	(2)
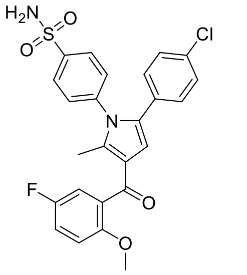	4-(5-(4-chlorophenyl)-3-(5-fluoro-2-methoxybenzoyl)-2-methyl-1H-pyrrol-1-yl)benzenesulfonamide	(3)
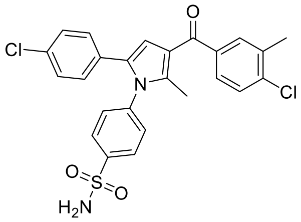	4-(3-(4-chloro-3-methylbenzoyl)-5-(4-chlorophenyl)-2-methyl-1H-pyrrol-1-yl)benzenesulfonamide	(3)

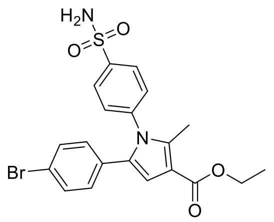	ethyl 5-(4-bromophenyl)-2-methyl-1-(4-sulfamoylphenyl)-1H-pyrrole-3-carboxylate	(3)
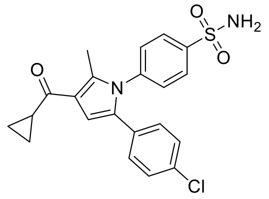	4-(5-(4-chlorophenyl)-3-(cyclopropanecarbonyl)-2-methyl-1H-pyrrol-1-yl)benzenesulfonamide	(3)
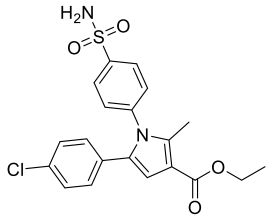	ethyl 5-(4-chlorophenyl)-2-methyl-1-(4-sulfamoylphenyl)-1H-pyrrole-3-carboxylate	(3)
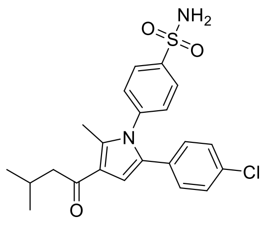	4-(5-(4-chlorophenyl)-2-methyl-3-(3-methylbutanoyl)-1H-pyrrol-1-yl)benzenesulfonamide	(3)
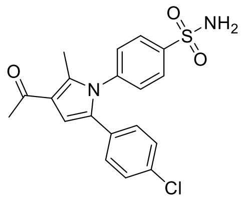	4-(3-acetyl-5-(4-chlorophenyl)-2-methyl-1H-pyrrol-1-yl)benzenesulfonamide	(3)
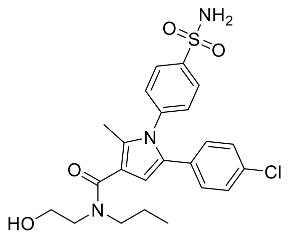	5-(4-chlorophenyl)-N-(2-hydroxyethyl)-2-methyl-N-propyl-1-(4-sulfamoylphenyl)-1H-pyrrole-3-carboxamide	(3)
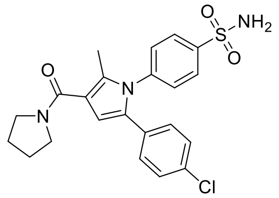	4-(5-(4-chlorophenyl)-2-methyl-3-(pyrrolidine-1-carbonyl)-1H-pyrrol-1-yl)benzenesulfonamide	(3)

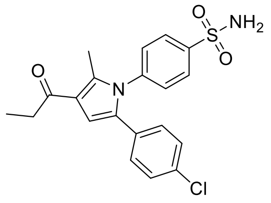	4-(5-(4-chlorophenyl)-2-methyl-3-propionyl-1H-pyrrol-1-yl)benzenesulfonamide	(3)
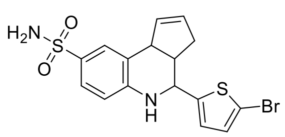	4-(5-bromothiophen-2-yl)-3a,4,5,9b-tetrahydro-3H-cyclopenta[c]quinoline-8-sulfonamide	(4)
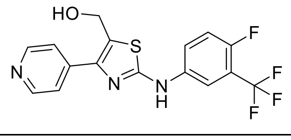	(2-((4-fluoro-3-(trifluoromethyl)phenyl)amino)-4-(pyridin-4-yl)thiazol-5-yl)methanol	(5)
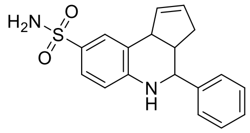	4-phenyl-3a,4,5,9b-tetrahydro-3H-cyclopenta[c]quinoline-8-sulfonamide	(2)
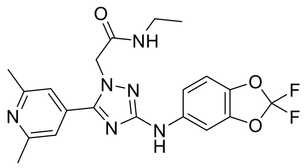	2-(3-((2,2-difluorobenzo[d][1,3]dioxol-5-yl)amino)-5-(2,6-dimethylpyridin-4-yl)-1H-1,2,4-triazol-1-yl)-N-ethylacetamide	(6)
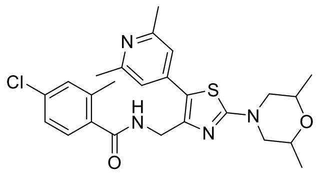	4-chloro-N-((2-(2,6-dimethylmorpholino)-5-(2,6-dimethylpyridin-4-yl)thiazol-4-yl)methyl)-2-methylbenzamide	(7)
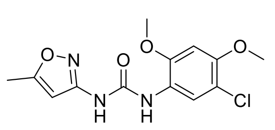	1-(5-chloro-2,4-dimethoxyphenyl)-3-(5-methylisoxazol-3-yl)urea	(8)
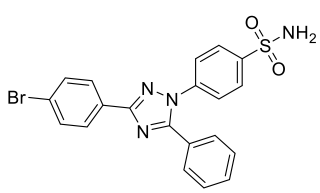	4-(3-(4-bromophenyl)-5-phenyl-1H-1,2,4-triazol-1-yl)benzenesulfonamide	(9)
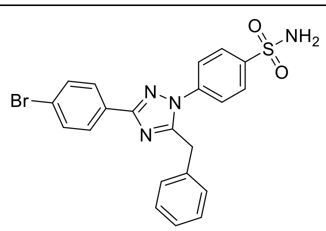	4-(5-benzyl-3-(4-bromophenyl)-1H-1,2,4-triazol-1-yl)benzenesulfonamide	(9)

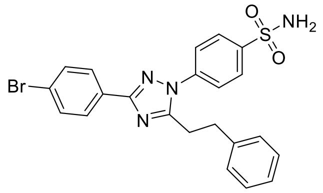	4-(3-(4-bromophenyl)-5-phenethyl-1H-1,2,4-triazol-1-yl)benzenesulfonamide	(9)

42 known active α7 nAChR type II positive allosteric modulators (PAMs) were used to create a database that was used for validation runs of the pharmacophore queries. Of these, 33 (all but nine) have been described previously in the literature and are included in this table.

1. Gill-Thind JK, Dhankher P, D'Oyley JM, Sheppard TD, Millar NS (2015) Structurally similar allosteric modulators of α7 nicotinic acetylcholine receptors exhibit five distinct pharmacological effects. *J Biol Chem* 290, 3552–3562.

2. Gill JK, Dhankher P, Sheppard TD, Sher E, Millar NS (2012) A series of α7 nicotinic acetylcholine receptor allosteric modulators with close chemical similarity but diverse pharmacological properties. *Mol Pharmacol* 81, 710–8.

3. Faghih R, et al. (2009) Discovery of 4-(5-(4-Chlorophenyl)-2-methyl-3-propionyl-1 H -pyrrol-1-yl)benzenesulfonamide (A-867744) as a novel positive allosteric modulator of the α7 nicotinic acetylcholine receptor. *J Med Chem* 52, 3377–3384.

4. Thakur GA, Kulkarni AR, Deschamps JR, Papke RL (2013) Expeditious synthesis, enantiomeric resolution, and enantiomer functional characterization of (4-(4-bromophenyl)-3a,4,5,9b-tetrahydro-3 h -cyclopenta[c]quinoline-8-sulfonamide (4BP-TQS): An allosteric agonist-positive allosteric modulator of alpha7 nAChR. *J Med Chem* 56, 8943–8947.

5. Dinklo T, et al. (2011) 4- (4-pyridinyl) -5-thiazolemethanol (JNJ-1930942), a novel positive allosteric modulator of the α7 nicotinic acetylcholine receptor. *Pharmacology* 1, 560–574.

6. Janssen Pharmaceutica NV; Thuring J, et al. (2007) 2-Aniline-4-aryl substituted thiazole derivatives; Patent WO 2009/115547 A1,

7. Janssen Pharmaceutica NV; Macdonald G. J., De Boeck B. C. A. G., Leenaerts J. E. (2011) Morpholinothiazoles as alpha 7 positive allosteric modulators; Patent WO 2011/064288 A1

8. Hurst RS, et al. (2005) A novel positive allosteric modulator of the alpha7 neuronal nicotinic acetylcholine receptor: in vitro and in vivo characterization. *J Neurosci* 25, 4396–4405.

9. Chatzidaki A, D'Oyley JM, Gill-Thind JK, Sheppard TD, Millar NS (2015) The influence of allosteric modulators and transmembrane mutations on desensitisation and activation of α7 nicotinic acetylcholine receptors. *Neuropharmacology* 97, 75–85.

Table S2. Validation data for pharmacophore queries

	AUC	0.5% enrichment	1% enrichment	2% enrichment
**Closed query 1**	0.96 ± 0.03	135.5 ± 24.9%	67.7 ± 12.4%	34.0 ± 6.5%
**Closed query 2**	0.97 ± 0.02	142.6 ± 23.2%	73.2 ± 12.6%	40.7 ± 7.6%
**Closed query 3**	0.96 ± 0.03	134.9 ± 27.4%	70.9 ± 13.3%	36.4 ± 6.2%
**Open query 1**	0.96 ± 0.03	136.1 ± 25.8%	70.6 ± 12.4%	36.8 ± 5.6%
**Open query 2**	0.97 ± 0.03	140.2 ± 26.2%	73.4 ± 11.9%	41.2 ± 5.7%
**Open query 3**	0.95 ± 0.03	135.7 ± 26.8%	67.8 ± 13.3%	33.9 ± 6.7%

Validation data for pharmacophore queries generated using vROCS v3.2.1.4

Table S3. Compounds identified by virtual screening

Rank	DrugBank ID	Compound Name	Target*
1	DB04763	1-N-(4-sulfamoylphenyl-ethyl)-2,4,6-trimethylpyridinium	CAII
2	DB07476	N-[4-(aminosulfonyl)phenyl]-2-mercaptobenzamide	CAII
3	DB08122	N-methyl-4-{[(2-oxo-1,2-dihydro-3h-indol-3-ylidene)methyl]amino}benzenesulfonamide	CDK2
4	DB08202	4-({[(4-methylpiperazin-1-yl)amino]carbonothioyl}amino)benzenesulfonamide	CAII
5	DB04371	AL6528	CAII
6	DB00695	Furosemide	NKCC
7	DB02602	AL7182	CAII
8	DB07257	4-(2-chlorophenyl)-8-(2-hydroxyethyl)-6-methylpyrrolo[3,4-e]indole-1,3(2H,6H)-dione	CAII
9	DB07048	N-[(2R)-5-(aminosulfonyl)-2,3-dihydro-1h-inden-2-yl]-2-propylpentanamide	CAII
10	DB02610	N-(2,3,4,5,6-pentaflouro-benzyl)-4-sulfamoyl-benzamide	CAII
11	DB03294	1-methyl-3-oxo-1,3-dihydro-benzo[c]isothiazole-5-sulfonic acid amide	CAII
12	DB01964	AL5424	CAII
13	DB02221	4-(aminosulfonyl)-N-[(2,4,6-trifluorophenyl)methyl]-benzamide	CAII
14	DB04549	4-(aminosulfonyl)-N-[(2,3,4-trifluorophenyl)methyl]-benzamide	CAII
15	DB03039	4-(aminosulfonyl)-N-[(2,5-difluorophenyl)methyl]-benzamide	CAII
16	DB04180	4-(aminosulfonyl)-N-[(2,4-difluorophenyl)methyl]-benzamide	CAII
17	DB03221	AL7099A	CAII
18	DB02220	AL7089A	CAII
19	DB07742	N-(2,3-difluoro-benzyl)-4-sulfamoyl-benzamide	CAII
20	DB03844	N-(2,6-diflouro-benzyl)-4-sulfamoyl-benzamide	CAII
21	DB02069	N-(2-flouro-benzyl)-4-sulfamoyl-benzamide	CAII
22	DB00487	Pefloxacin	DNAG
23	DB03333	(4-sulfamoyl-phenyl)-thiocarbamic acid O-(2-thiophen-3-yl-ethyl) ester	CAII
24	DB03950	(S)-N-(3-indol-1-yl-2-methyl-propyl)-4-sulfamoyl-benzamide	CAII
25	DB02479	(R)-N-(3-indol-1-yl-2-methyl-propyl)-4-sulfamoyl-benzamide	CAII
26	DB07791	4-{[4-(1-cyclopropyl-2-methyl-1h-imidazol-5-yl)pyrimidin-2-yl]amino}-N-methylbenzenesulfonamide	CDK2
27	DB08673	4-[(5-isopropyl-1,3-thiazol-2-yl)amino]benzenesulfonamide	CDK2
28	DB08083	2-(1,3-thiazol-4-yl)-1h-benzimidazole-5-sulfonamide	CAII
29	DB08165	indane-5-sulfonamide	CAII
30	DB07798	(3R)-3-(fluoromethyl)-N-(3,3,3-trifluoropropyl)-1,2,3,4-tetrahydroisoquinoline-7-sulfonamide	PNMT
31	DB06771	Besifloxacin	DNAG
32	DB07115	N-(4-chlorobenzyl)-N-methylbenzene-1,4-disulfonamide	CAXIII
33	DB07363	Thiophene-2,5-disulfonic acid 2-amide-5-(4-methyl-benzylamide)	CAII
34	DB08134	4-[(6-chloropyrazin-2-yl)amino]benzenesulfonamide	CDK2
35	DB01208	Sparfloxacin	DNAG
36	DB01689	Inhibitor Idd 384	AR
37	DB04608	9-hydroxy-4-phenyl-6h-pyrrolo[3,4-c]carbazole-1,3-dione	W1LPK
38	DB02292	Irosustat	CAII
39	DB02197	4-[(4-imidazo[1,2-a]pyridin-3-ylpyrimidin-2-yl)amino]benzenesulfonamide	CDK2
40	DB01059	Norfloxacin	DNAG
41	DB00817	Rosoxacin	DNAG
42	DB08301	N-({[4-(aminosulfonyl)phenyl]amino}carbonyl)-4-methylbenzenesulfonamide	CAII
43	DB02741	CD564	RAR
44	DB05488	Technetium Tc-99m ciprofloxacin	DNAG
45	DB00537	Ciprofloxacin	DNAG
46	DB01657	2-amino-3-[4-hydroxy-6-oxo-3-(2-phenyl-cyclopropylimino)-cyclohexa-1,4-dienyl]-propionic acid	PAO
47	DB03034	D-Levofloxacin	DNAG
48	DB04089	AL5300	CAII
49	DB07226	N-[4-(2-chlorophenyl)-1,3-dioxo-1,2,3,6-tetrahydropyrrolo[3,4-c]carbazol-9-yl]formamide	W1LPK
50	DB08106	N-[(6-butoxynaphthalen-2-yl)sulfonyl]-D-glutamic acid	UDGL
51	DB08105	N-[(6-butoxynaphthalen-2-yl)sulfonyl]-L-glutamic acid	UDGL
52	DB01224	Quetiapine	DR
53	DB00311	Ethoxzolamide	CAII
54	DB01137	Levofloxacin	DNAG
55	DB08157	Ethyl 3-[4-(aminosulfonyl)phenyl]propanoate	CAII
56	DB07006	9-hydroxy-6-(3-hydroxypropyl)-4-(2-methoxyphenyl)pyrrolo[3,4-c]carbazole-1,3(2h,6h)-dione	W1LPK
57	DB00467	Enoxacin	DNAG
58	DB08107	N-{[6-(pentyloxy)naphthalen-2-yl]sulfonyl}-d-glutamic acid	UDGL
59	DB01194	Brinzolamide	CAII
60	DB09047	Finafloxacin	DNAG
61	DB00218	Moxifloxacin	DNAG
62	DB02861	4-(aminosulfonyl)-N-[(3,4,5-trifluorophenyl)methyl]-benzamide	CAII
63	DB07790	N-(2-methoxyethyl)-4-({4-[2-methyl-1-(1-methylethyl)-1h-imidazol-5-yl]pyrimidin-2-yl}amino)benzenesulfonamide	CDC2
64	DB07265	3-(9-hydroxy-1,3-dioxo-4-phenyl-2,3-dihydropyrrolo[3,4-c]carbazol-6(1h)-yl)propanoic acid	W1LPK
65	DB11491	Sarafloxacin	DNAG
66	DB08304	(3r)-3-cyclopentyl-7-[(4-methylpiperazin-1-yl)sulfonyl]-3,4-dihydro-2h-1,2-benzothiazine 1,1-dioxide	GR
67	DB05095	Cimicoxib	COX

68	DB08303	(3S)-3-cyclopentyl-6-methyl-7-[(4-methylpiperazin-1-yl)sulfonyl]-3,4-dihydro-2H-1,2,4-benzothiadiazine 1,1-dioxide	GR
69	DB06803	Niclosamide	RT
70	DB08729	5-ethoxy-4-(1-methyl-7-oxo-3-propyl-6,7-dihydro-1H-pyrazolo[4,3-d]pyrimidin-5-yl)thiophene-2-sulfonamide	CSCP
71	DB06891	5-{[(4-amino-3-chloro-5-fluorophenyl)sulfonyl]amino}-1,3,4-thiadiazole-2-sulfonamide	CAII
72	DB08701	2-(3-bromophenyl)-6-[(2-hydroxyethyl)amino]-1h-benzo[de]isoquinoline-1,3(2h)-dione	GP
73	DB07050	5-[(phenylsulfonyl)amino]-1,3,4-thiadiazole-2-sulfonamide	CAII
74	DB02429	4-(aminosulfonyl)-n-[(4-fluorophenyl)methyl]-benzamide	CAII
75	DB00998	Frovatriptan	SR
76	DB00685	Trovafloxacin	DNAG
77	DB08485	(1S,4S,5S)-1,4,5-trihydroxy-3-[3-(phenylthio)phenyl]cyclohex-2-ene-1-carboxylic acid	DD
78	DB01748	N-benzyl-4-sulfamoyl-benzamide	CAII
79	DB03307	4-[(6-amino-4-pyrimidinyl)amino]benzenesulfonamide	CDK2
80	DB03873	Tamibarotene	RAR
81	DB07683	N-(dibenzo[b,d]thiophen-3-ylsulfonyl)-L-valine	MM

Compounds identified by virtual screening of the DrugBank database are listed in rank order. The four compounds examined by pharmacological techniques are highlighted in red.

* Target site of compounds identified in the DrugBank database. Abbreviations used:
AR: aldose reductase
CAII: carbonic anhydrase II
CAXIII: carbonic anhydrase XIII
CDK2: cyclin-dependent kinase 2
COX: cyclooxygenase
CSCP: cGMP-specific 3',5'-cyclic phosphodiesterase
DNAG: DNA gyrase/topoisomerase (target of fluoroquinolone antibiotics)
DD: 3-dehydroquinate dehydratase
DR: dopamine receptor
GP: genome polyprotein
GR: glutamate receptor
MM: macrophage metalloelastase
NKCC: $Na^+/K^+/2Cl^-$ cotransporter (target of loop diuretics)
PAO: primary-amine oxidase
PNMT: phenylethanolamine N-methyltransferase
RAR: retinoic acid receptor
RT: regulation of transcription
SR: serotonin receptor
UDGL: UDP-N-acetylmuramoylalanine-D-glutamate ligase
W1LPK: Wee1-like protein kinase

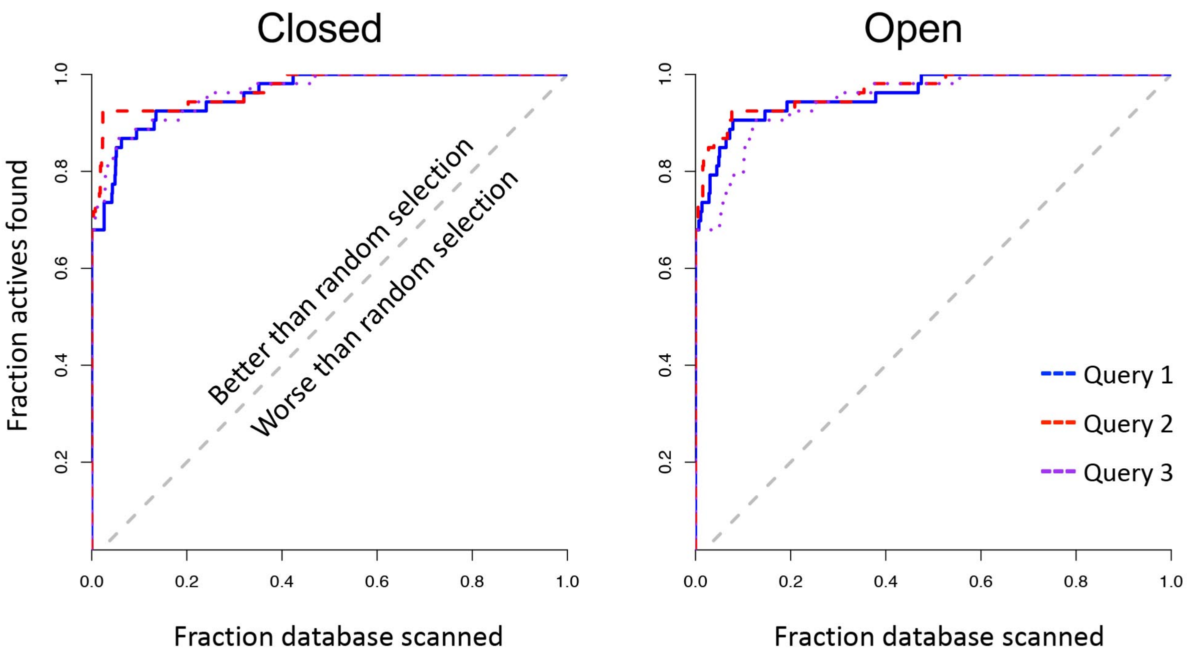

Figure S1

Validation statistics of pharmacophore queries used for virtual screening. Receiver operator characteristic (ROC) plots for closed conformation (left) and open conformation (right) screenings. The dashed grey diagonal line shows AUC = 0.5, where queries above this line are better than random selection at choosing genuine hits over decoys, and queries below are worse than random selection at choosing genuine hits over decoys. For both conformation screenings, query 1 is shown as a solid blue line, query 2 as dashed red line and query 3 as a dashed purple line.

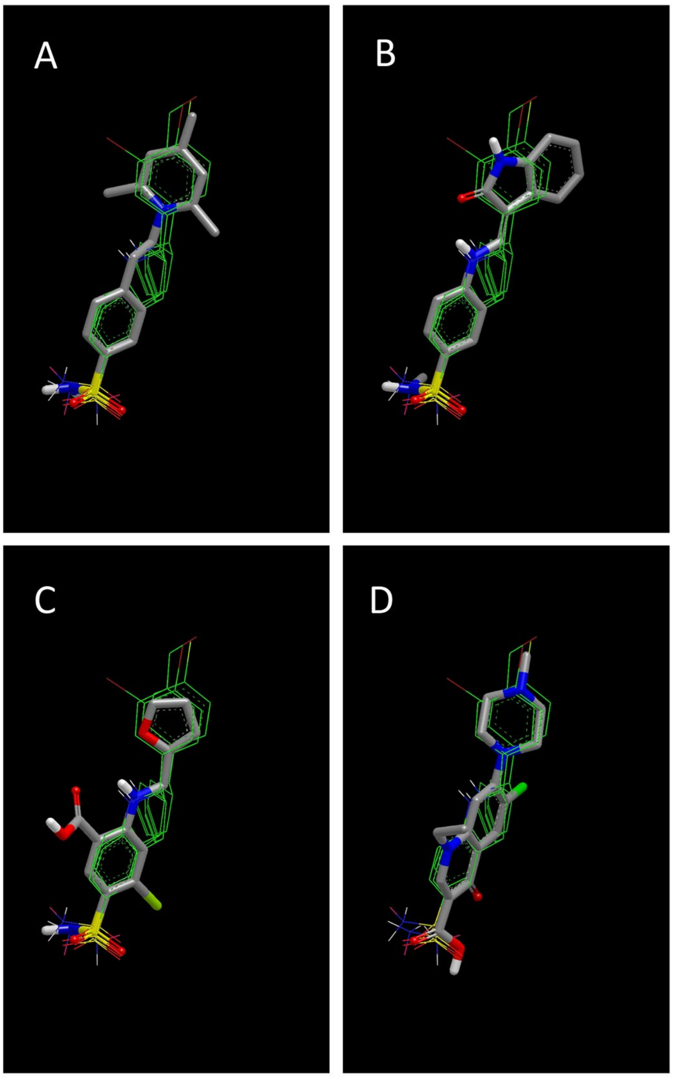

Figure S2

Virtual screening ligand overlays. The structure of DB04763 (A) , DB08122 (B), furosemide (C) and pefloxacin (D) are shown (in stick form) overlaid on the molecules used to create the pharmacophore query (shown in wireframe). Atoms are represented as follows: carbon (grey), oxygen (red), nitrogen (blue), sulfur (yellow), chlorine (light green), fluorine (green) and hydrogen (white).

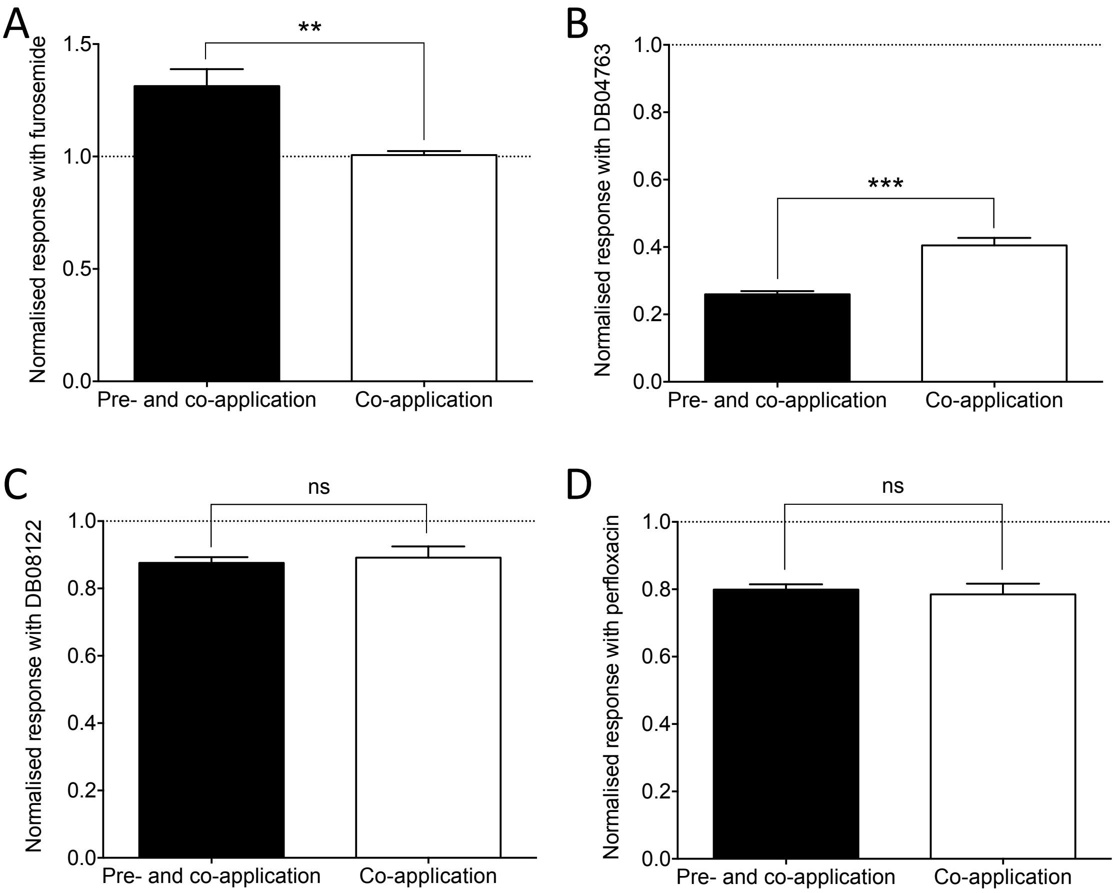

Figure S3

The influence of pre-application of allosteric modulators on agonist-evoked responses on α7 nAChRs. Responses to a submaximal (EC_{50}) concentration of ACh (100 μM) were determined on wild-type α7 nAChRs expressed in Xenopus oocytes. The magnitude of responses were compared to that observed in the presence furosemide (A), DB04763 (B), DB08122 (C) and pefloxacin (D). Test compounds were either pre-applied for 30 s, followed by coapplication with agonist, or were co-applied without pre-application. Responses are normalised to that observed in the absence of test compounds. Data are means ± SEM (n = 4-7). Significant differences between responses in the presence or absence of pre-application are indicated (** P <0.01, *** P < 0.001).
